# Analysis of prognostic factors and risk prediction in brain metastases: a SEER population-based study

**DOI:** 10.3389/fonc.2025.1523069

**Published:** 2025-05-21

**Authors:** Qiang Ji, Zixuan Yang, Xun Kang, Lili Zhou, Feng Chen, Wenbin Li

**Affiliations:** ^1^ Department of Neuro-oncology, Cancer Center, China National Clinical Research Center for Neurological Diseases, Beijing Tiantan Hospital, Capital Medical University, Beijing, China; ^2^ National Institute for Data Science in Health and Medicine, Capital Medical University, Beijing, China

**Keywords:** brain metastases, risk factors, prediction model, SEER, prognosis

## Abstract

**Background:**

This study investigates survival disparities and prognostic factors in patients with brain metastases originating from various primary cancers to facilitate risk stratification and enhance precision in diagnosis and treatment.

**Methods:**

Patients diagnosed with brain metastases between 2010 and 2018 were identified from the SEER database for analysis. Overall survival (OS) was evaluated using Kaplan-Meier curves and log-rank tests, complemented by multivariate Cox regression analysis. The impact of age on the risk and survival of brain metastases was examined using Restricted Cubic Splines (RCS) in Cox regression models.

**Results:**

A total of 55,094 patients diagnosed with brain metastases between 2010 and 2018 were retrospectively identified from the SEER database for inclusion in this study. It was found that the median survival times were 2 months (95% CI: 2–3 months) for liver cancer, 3 months (95% CI: 3–4 months) for stomach cancer, and 5 months (95% CI: 4–5 months) for lung cancer. Survival was influenced by factors such as sex, age, primary cancer site, race, income, marital status, and treatment approaches. Surgical treatment notably decreased the mortality risk, with a hazard ratio (HR) of 0.49 (95% CI: 4–5 months) for lung cancer, 0.43 (95% CI:3–4 months) for kidney cancer, and 0.63 (95% CI: 5–7 months) for breast cancer. The predictive model created with these variables achieved a C-index of 0.723 and 0.722 in the training and test sets, respectively, indicating vital accuracy. Calibration curves displayed minimal errors, and the area under the curve (AUC) values showed excellent performance at 3 months (training: 0.83, test: 0.83), 6 months (training: 0.80, test: 0.80), and 12 months (training: 0.77, test: 0.76).

**Conclusion:**

Brain metastases from liver, stomach, and lung cancers are linked to a poor prognosis. Surgical intervention significantly lowers mortality risk. The predictive model, which incorporates vital survival factors, demonstrates high accuracy and reliable performance, supporting the clinical management of patients with brain metastases.

**Systematic Review Registration:**

https://www.crd.york.ac.uk/prospero, identifier CRD420251054176.

## Introduction

Approximately 20% of cancer patients are diagnosed with brain metastases during their disease course (1–3), though this estimate is likely conservative. Autopsy studies suggest a higher incidence, with brain metastases identified in 30–40% of cancer patients (4, 5). Primary tumours most commonly linked to brain metastases include lung cancer, which affects 20–56% of these patients, breast cancer (5–20%), and melanoma (7–16%) (6–8). The presence of brain metastases is typically indicative of advanced disease and is correlated with a poor prognosis (8–11). For individuals diagnosed with brain metastases, OS rates are alarmingly low, with only 5-24% surviving up to two years and a mere 2.4-15% reaching the five-year mark, regardless of the type of primary tumour (12–15). In addition to sex, tumour origin, and molecular subtype, the development of brain metastases is also affected by ethnicity, geographic location, age, and treatment methods (1). A comprehensive examination of these known and unidentified factors that may influence the occurrence and prognosis of brain metastases could significantly improve clinical management and enhance treatment outcomes for affected patients (16). For instance, research conducted by Kuksis et al. has shown a high prevalence of brain metastases in patients with HER2-positive and triple-negative metastatic breast cancer (MBC). Improving screening for brain metastases in patients with HER2-positive and triple-negative MBC could enable earlier detection and treatment, ultimately enhancing therapeutic outcomes (17). Additionally, Tsai et al. found that survival was significantly reduced in patients with brain metastases originating from gastroesophageal adenocarcinoma who did not receive surgery or radiotherapy, according to multivariable analyses (14).

Furthermore, research conducted by K. Salari et al. has demonstrated that, in patients with brain-only metastatic non-small cell lung cancer (NSCLC), definitive treatment of the thoracic primary site after intracranial radiosurgery was linked to slower disease progression and improved survival (18). These studies offer valuable insights and recommendations for treating brain metastases. Still, they focus exclusively on metastases from specific primary sites without comprehensively analysing those originating from various primary locations. The research by W. Sperduto et al. addresses this gap by performing a multifactorial analysis of factors that influence prognosis in individuals with brain metastases, which led to the development of the Graded Prognostic Assessment (GPA) for brain metastases from multiple primary sites (19). This allows for risk stratification and treatment guidance using the GPA, effectively overcoming the limitations of the studies mentioned above. However, when constructing the GPA, the researchers did not consider the primary site as a covariate, instead opting for a generalized analysis. This approach is inadequate because prognoses vary significantly based on the primary site (20, 21). In the latest iteration of the GPA (22), the authors introduced stratification to mitigate the influence of the primary site. However, this approach created separate scoring scales for each type of brain metastasis, rendering the GPA calculation overly complex.

This study aims to utilize data from patients with brain metastases originating from various primary sites to develop a tool for risk stratification. By analysing relevant prognostic factors, the study seeks to assist clinicians in delivering precise diagnoses and tailored treatments for patients affected by brain metastases.

## Materials and methods

### Data extraction

Study data were extracted using SEER*Stat software (version 8.3.9), utilising the “Incidence-SEER Research Plus Data, 18 Registries, Nov 2020 Sub (2000–2018)” dataset. All cases were initially identified using International Classification of Diseases for Oncology, Third Edition (ICD-O-3) histology and site codes relevant to the study population.

Due to the absence of the “SEER Combined Mets at DX-brain” variable for patients diagnosed before 2010, it was impossible to determine the presence of brain metastases in these cases. Therefore, the study included only patients diagnosed with brain metastases between 2010 and 2018. Those with missing data on age at diagnosis, race, sex, marital status, or incomplete follow-up were excluded. The primary endpoint of this study was OS, defined as the time from diagnosis to death from any cause or last follow-up.

### Study design

**Figure 1:**
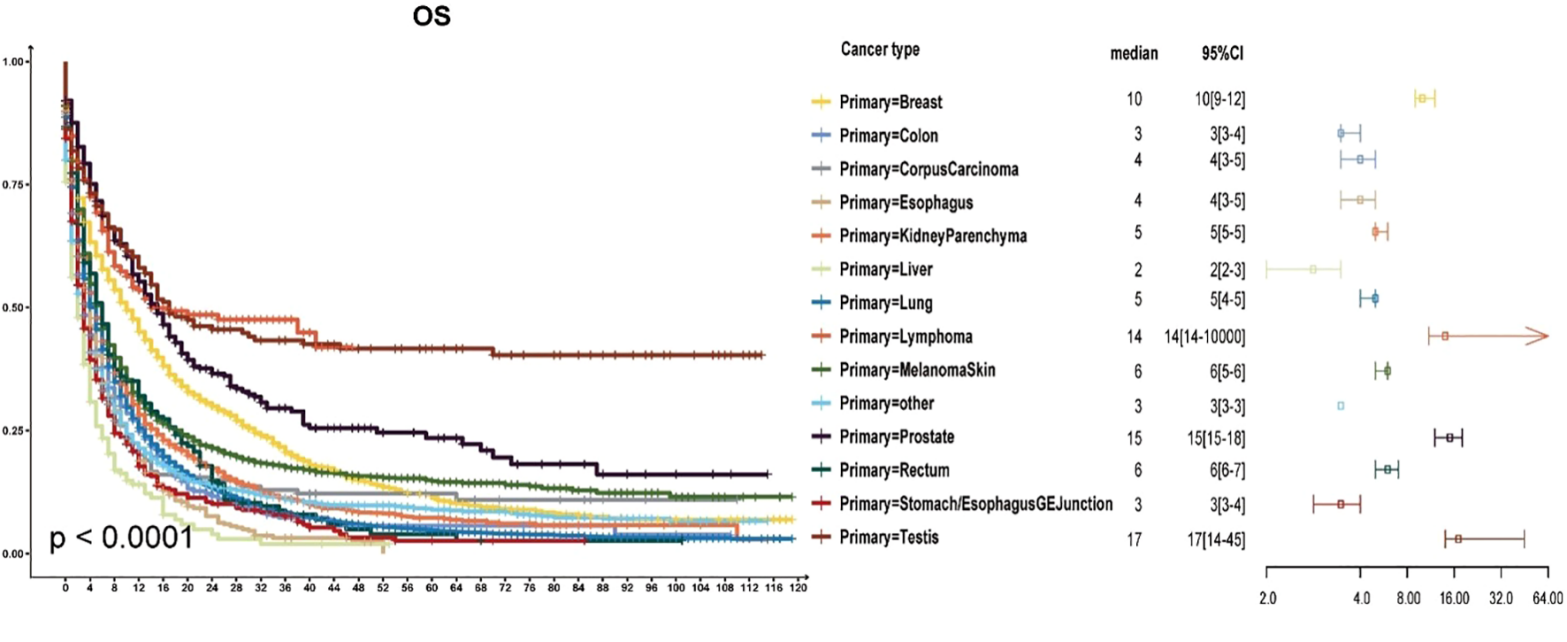
Survival curves for different primary sites of brain metastases.

The overall study design is illustrated in a flowchart (**Figure 1**). A total of 55,094 patients diagnosed with brain metastases between 2010 and 2018 were retrospectively identified from the SEER database. After data cleaning and selection based on predefined inclusion and exclusion criteria, eligible patients were randomly assigned to training and testing sets in a 7:3 ratio. A multivariable Cox regression model was applied to identify independent prognostic factors, and a nomogram was constructed based on variables including age, sex, race, income, marital status, primary site, surgery, radiotherapy, and chemotherapy to predict 1-year OS. The performance of the nomogram was assessed using the concordance index (C-index), time-dependent Receiver Operating Characteristic (ROC) curves, and calibration plots at 3, 6, and 12 months in both training and testing sets. The total nomogram score for each patient was calculated, and the corresponding AUC was used to compare the predictive accuracy of the nomogram with the TNM clinical staging system.

### Statistical analysis

The primary endpoint of this study was OS. Categorical variables included sex, race (White, Black, Other), and marital status (married, single, divorced/separated, widowed). Age was treated both as a categorical variable (using clinically meaningful age groups) and as a continuous variable in selected models.

Kaplan-Meier curves and the log-rank test were used to assess the survival rate. To evaluate prognostic factors associated with OS, univariate Cox proportional hazards regression was first performed. Variables with p < 0.05 in univariate analysis were included in a multivariate Cox regression model to identify independent predictors of survival. HR and 95% confidence interval (CI) were reported.

The effect of age on the likelihood of developing brain metastases and related survival outcomes was analysed continuously through restricted cubic splines RCS within Cox regression models, with three to five knots placed at percentiles of the age distribution. Model performance was evaluated using the concordance index (C-index) to assess discriminative ability, and time-dependent ROC curves were utilized to evaluate predictive accuracy at different time points (e.g., 3, 6, 12 months). Calibration plots comparing predicted versus observed survival probabilities were used to assess model calibration.

All statistical analyses were carried out using R software (version 4.3.1) with relevant packages including “survival”, “rms”, “timeROC”, and “ggplot2”.

## Results

### Patient characteristics

55,094 patients diagnosed with brain metastases between 2010 and 2018 were enrolled in this study (**Table 1**). The survival distribution of the patients followed a U-shaped pattern, with a higher proportion of patients having survival times of less than six months or more significant than twelve months. Patients having survival times of between six and 12 months were less common. Specifically, 59.1% of patients with brain metastases had an OS of less than six months, including 4.2% who did not reach the endpoint. In contrast, 22.8% of the population had survival times exceeding 12 months. Among patients with survival times less than 12 months, the proportion of males was higher than females. However, the trend reversed in those with survival times more significant than 12 months, with a significantly higher proportion of females (p < 0.001).

**Table 1:** Clinicopathologic characteristics associated with OS five.

Characteristics	0–6 months	6–12 months	>12 months	p
Overall	32600	9940	12554	
Sex				<0.001
Female	14651 (44.9)	4782 (48.1)	6807 (54.2)	
Male	17949 (55.1)	5158 (51.9)	5747 (45.8)	
Primary				<0.001
Breast	892 (2.7)	300 (3.0)	763 (6.1)	
Colon	355 (1.1)	85 (0.9)	123 (1.0)	
Endometrial Carcinoma	163 (0.5)	47 (0.5)	36 (0.3)	
Esophagus	360 (1.1)	103 (1.0)	88 (0.7)	
KidneyParenchyma	1002 (3.1)	314 (3.2)	408 (3.2)	
Liver	154 (0.5)	25 (0.3)	17 (0.1)	
Lung	25952 (79.6)	8052 (81.0)	9645 (76.8)	
Lymphoma	112 (0.3)	55 (0.6)	95 (0.8)	
MelanomaSkin	1085 (3.3)	374 (3.8)	512 (4.1)	
other	1975 (6.1)	408 (4.1)	483 (3.8)	
Prostate	122 (0.4)	65 (0.7)	153 (1.2)	
Rectum	133 (0.4)	38 (0.4)	70 (0.6)	
Stomach/EsophagusGEJunction	224 (0.7)	48 (0.5)	54 (0.4)	
Testis	71 (0.2)	26 (0.3)	107 (0.9)	
Race				<0.001
Black	3813 (11.7)	1168 (11.8)	1307 (10.4)	
Chinese	420 (1.3)	196 (2.0)	412 (3.3)	
Other	2193 (6.7)	682 (6.9)	1399 (11.1)	
Unknown	91 (0.3)	32 (0.3)	39 (0.3)	
White	26083 (80.0)	7862 (79.1)	9397 (74.9)	
Age				<0.001
mean (sd)	66.41 (10.97)	62.79 (11.28)	60.41 (12.18)	
Income				<0.001
<35000	935 (2.9)	280 (2.8)	240 (1.9)	
35000-45000	3316 (10.2)	937 (9.4)	942 (7.5)	
45000-55000	5677 (17.4)	1666 (16.8)	1785 (14.2)	
55000-65000	7236 (22.2)	2133 (21.5)	2664 (21.2)	
65000-75000	6776 (20.8)	2108 (21.2)	2682 (21.4)	
>75000	8660 (26.6)	2814 (28.3)	4241 (33.8)	
Unknown	0 (0.0)	2 (0.0)	0 (0.0)	
Marital				<0.001
Married	14931 (45.8)	5129 (51.6)	7038 (56.1)	
Other	9588 (29.4)	2476 (24.9)	2678 (21.3)	
Single	6636 (20.4)	1922 (19.3)	2380 (19.0)	
Unknown	1445 (4.4)	413 (4.2)	458 (3.6)	
Surgery				<0.001
No/unknown	30755 (94.3)	9264 (93.2)	11114 (88.5)	
Yes	1845 (5.7)	676 (6.8)	1440 (11.5)	
Chemotherapy				<0.001
No/Unknown	22282 (68.3)	2407 (24.2)	2716 (21.6)	
Yes	10318 (31.7)	7533 (75.8)	9838 (78.4)	
Radiation				<0.001
No/unknown	14224 (43.6)	2078 (20.9)	2612 (20.8)	
Yes	18376 (56.4)	7862 (79.1)	9942 (79.2)	

Among all primary sites of brain metastases, lung cancer had the highest incidence (79.2%), followed by skin cancer (3.8%), breast cancer (3.5%), and kidney parenchyma cancer (3.1%). At the initial diagnosis of brain metastases, a younger age was associated with a better subsequent prognosis. The mean age at initial diagnosis for patients with survival times of 0–6 months was 66.41 years. The mean ages for the better survival groups were significantly lower, at 62.79 and 60.41 years, respectively.

In addition to the factors above of age, sex, and primary site, other variables such as race, income, marital status, and treatment modalities also significantly influenced the prognosis of brain metastases. All observed differences were statistically significant (p < 0.001). However, due to the retrospective nature of the SEER database, certain data points may be incomplete or missing, potentially impacting the accuracy and generalizability of these findings.

The p-values in **Table 1** were calculated based on grouping the population into three categories according to survival time. For categorical variables, we applied the chi-square test; for continuous variables, one-way ANOVA was used. When the assumptions of the chi-square test or ANOVA were not met, non-parametric tests were employed to assess differences among the survival groups.

### Survival analysis

As shown in **Figure 1**, most patients with brain metastases from various primary sites had a median survival time of less than six months. The poorest prognosis was observed in patients with brain metastases from liver cancer, where the median survival time was two months (95% CI: 2–3 months). Brain metastases from stomach cancer had a median survival time of three months (95% CI: 3–4 months). Lung cancer, which represented the most significant proportion of brain metastases, also had a poor prognosis with a median survival time of five months (95% CI: 4–5 months). Conversely, patients with brain metastases from breast cancer, lymphoma, prostate cancer, and testicular cancer had a median survival time exceeding ten months. Among these, testicular cancer had the most favourable prognosis, with a median survival time surpassing 17 months (95% CI: 14–45 months).

In addition to the primary site of origin, age is a critical factor influencing the prognosis of patients with brain metastases. Cancer is often considered a disease of aging, with its occurrence and outcomes closely linked to the aging process (23). We employed RCS to model these effects and explore further the relationship between the primary site of brain metastases and the age-related risk variation in survival.RCS is a flexible statistical method for modelling non-linear relationships between variable (24). By applying RCS in our analysis, we were able to examine how the risk of mortality changes with age among patients with brain metastases from different primary cancers. This approach provides a nuanced understanding of the impact of age on survival, taking into account the varying risks associated with other primary sites.

**Figure 2:**
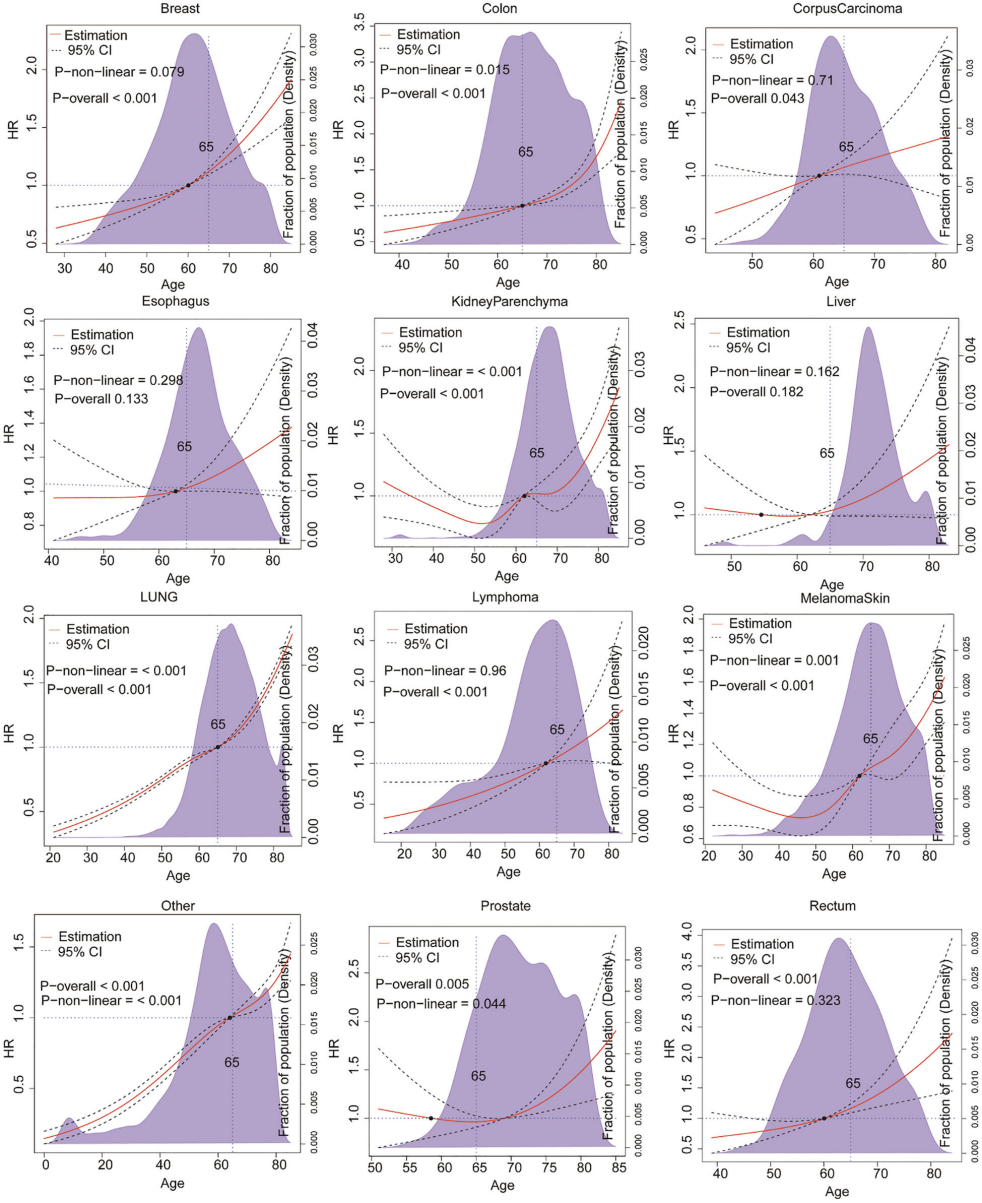
Age-related risk variation in brain metastases from different primary sites.

As illustrated in **Figure 2**, the findings of our study indicate that the risk of survival following the development of brain metastases from the majority of primary sites increases with age. However, the pattern of this age-related risk varies among different primary cancers. For patients with brain metastases originating from lung cancer, breast cancer, and lymphoma, the mortality hazard exhibits a rapid increase with age across all age groups (P < 0.001). In contrast, for patients with brain metastases from testicular cancer, colorectal cancer, and EndometrialCarcinoma, the risk of mortality due to brain metastases remains relatively stable until the age of 65, after which it increases sharply. It is noteworthy that patients with brain metastases from liver cancer and rectal cancer also exhibit an age-related increase in mortality risk. However, this trend does not reach statistical significance (P>0.05). These findings emphasise the necessity of considering both the primary site of cancer and the patient’s age when evaluating prognosis and developing treatment strategies for brain metastases.

**Figure 3:**
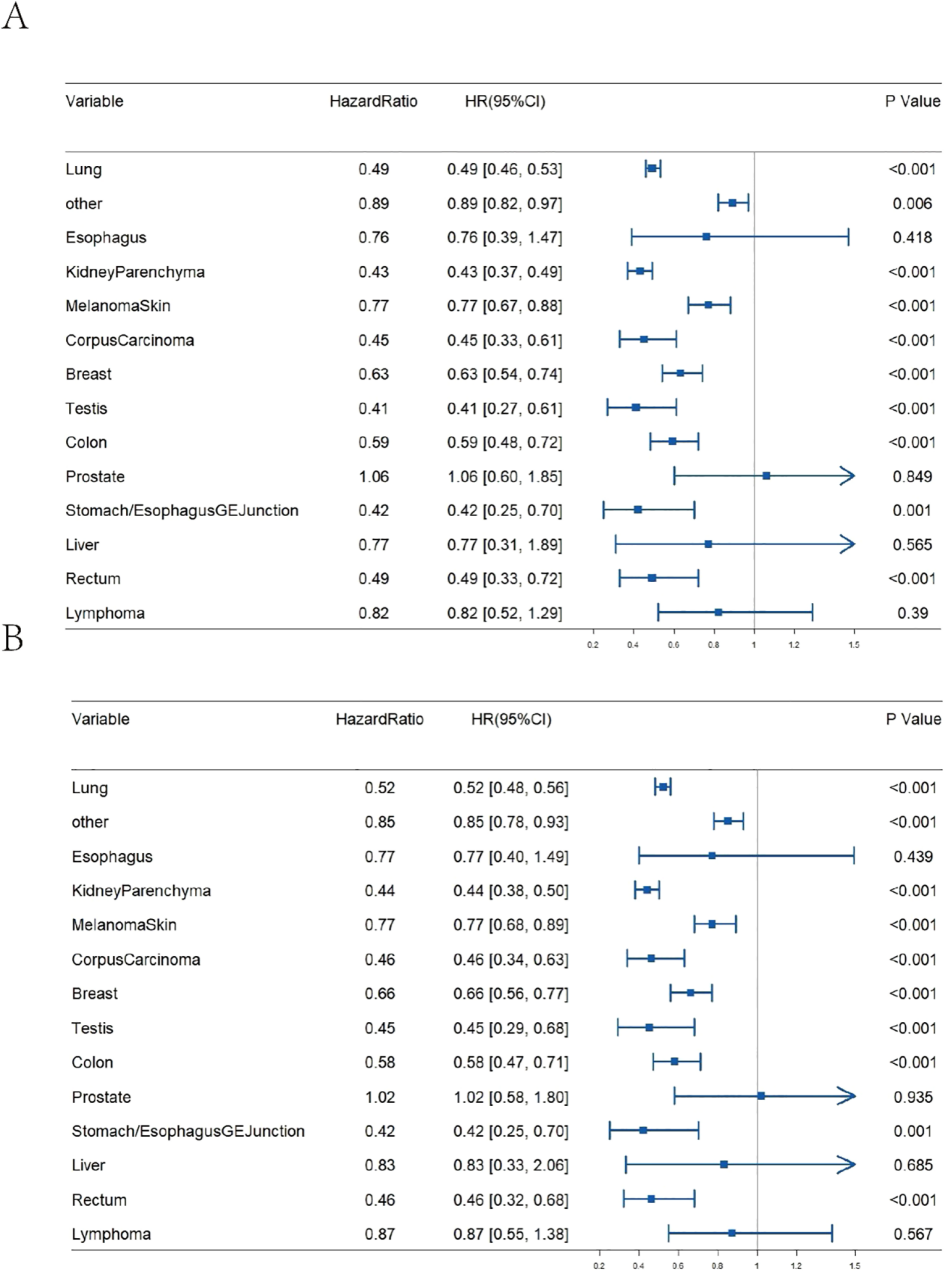
Impact of surgical resection on OS for brain metastases from various primary tumour sites. **(A)** Un-adjusted age and gender; **(B)** Adjusted age and gender.

The decision between radiotherapy and surgical resection (”surgical resection” in this manuscript specifically refers to neurosurgical resection of metastatic brain lesions, not resection of the primary tumours.)can be influenced by several factors, including the size and diversity of the tumour (25–27). While the majority of contemporary studies concentrate on tumour size to ascertain the necessity of surgery for brain metastases, there has been a lack of research investigating whether the primary location of the tumour influences the decision to operate. As illustrated in **Figure 3**, the results of our study indicate that surgical intervention for brain metastases originating from various primary sites generally reduces the risk of death. For instance, the mortality risk for lung cancer metastases following surgery is reduced by nearly half, with an HR of 0.49 [95% CI 0.46-0.53]. After adjusting for age and gender, the HR is 0.52 [95% CI 0.48-0.56]. Similarly, the HR for kidney parenchyma metastases is 0.43 [95% CI 0.37-0.49] and 0.44 [95% CI 0.38-0.50] after adjustment. The HR for breast cancer metastases is 0.63 [95% CI 0.54-0.74] and 0.66 [95% CI 0.56-0.77] after adjustment. Nevertheless, it should be noted that not all tumours with brain metastases are suitable for surgical treatment. For instance, patients with brain metastases from liver cancer, bladder cancer, or esophageal cancer do not exhibit a significant survival benefit post-surgery (P>0.05).

### Development and validation of a survival prediction model for patients with brain metastases

**Figure 4:**
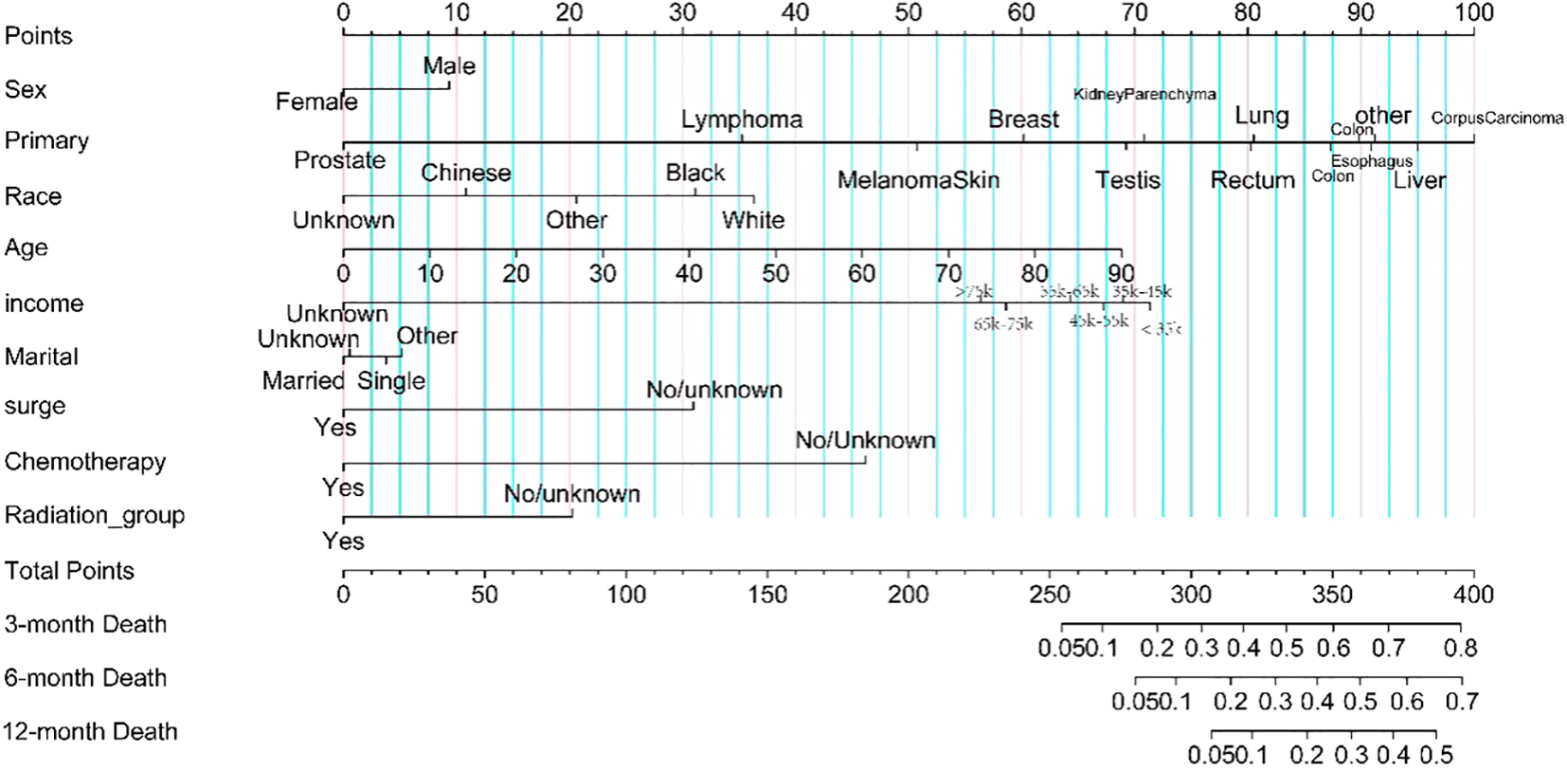
Nomogram for the prediction of survival rate in brain metastases.

**Figure 5:**
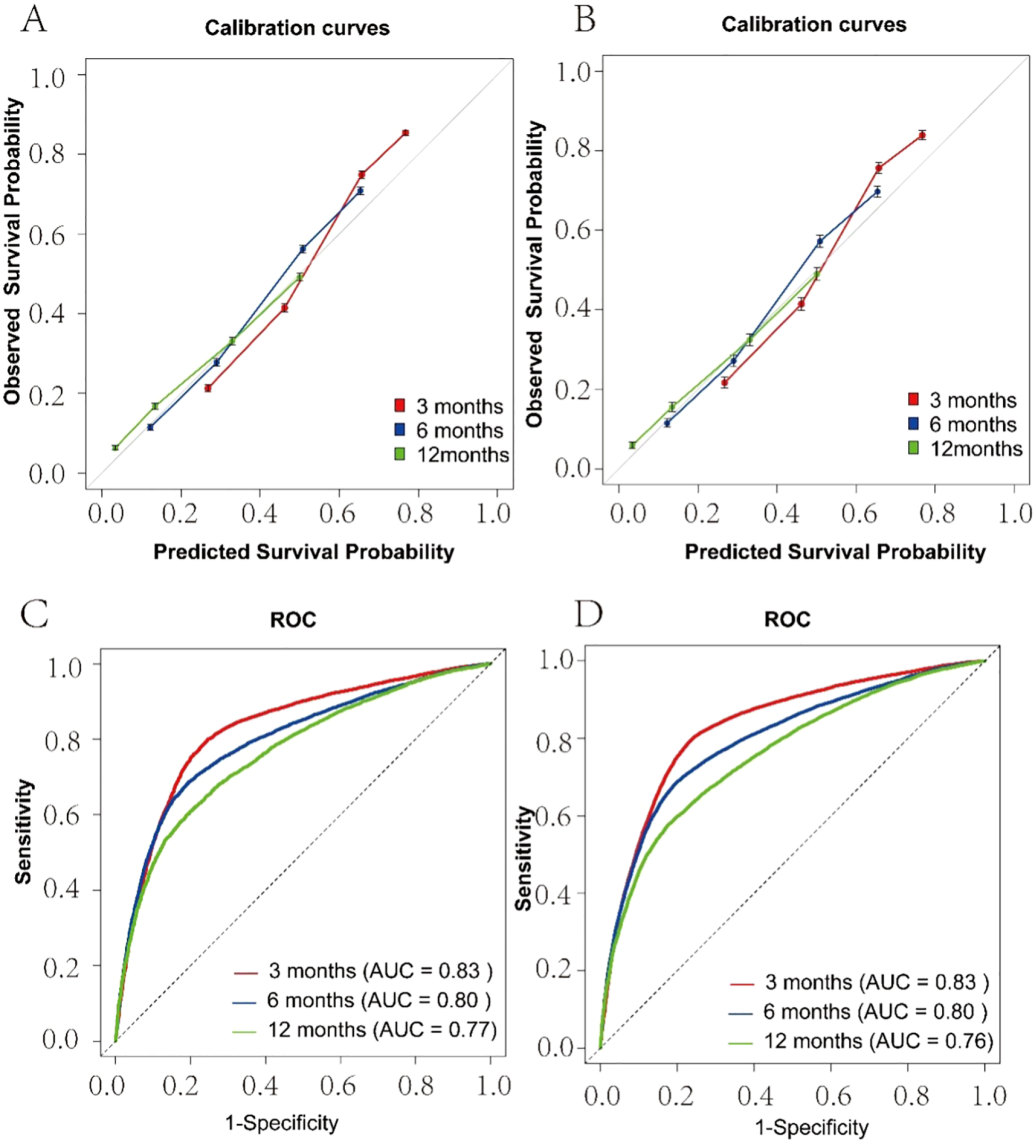
Calibration Plot and ROC for the Prediction of 3,6,12-months Survival Rate. **(A)** Training Set Calibration Curve; **(B)** Test Set Calibration Curve; **(C)** Training Set ROC; **(D)** Test Set ROC.

Seventy percent of patients with brain metastases were randomly assigned to the training set, while 30% were assigned to the test set. The prediction model incorporated variables related to survival, such as sex, age, race, income, marital status, surgery, radiotherapy, and chemotherapy. To enhance usability, we created a nomogram (see **Figure 4**) that assesses the importance of these variables in predicting patient survival. Higher scores indicate a more significant impact on survival. The model’s robustness was evaluated using the C-index, ROC, and calibration curves. It showed excellent performance in both sets, with calibration curves in **Figures 5A, B** indicating that predicted survival values at 3, 6, and 12 months closely matched observed values, reflecting high stability. The C-index was 0.723 for the training set and 0.722 for the test set. The time-dependent AUC for the training set was 0.83, 0.80, and 0.77 for the respective time points, while the test set yielded values of 0.83, 0.80, and 0.76, as shown in **Figures 5C, D**.

## Discussion

Brain metastases are the most prevalent form of intracranial tumour (28). Historically, the prognosis for brain metastasis has been poor, with over half of the patients dying within 3 to 27 months of diagnosis (6, 29). This is consistent with our study findings, which underscore the significant burden brain metastases impose on patient survival. It is, therefore, crucial to analyse and explore the factors influencing survival in this context to inform subsequent treatments and reduce patient mortality risk. However, current research on brain metastasis primarily focuses on metastasis from a single primary site or lacks a practical tool for timely assessment of mortality hazard in patients with brain metastasis. Our study comprehensively compared risk factors for patients with brain metastases from different primary sites. This allowed us to identify commonalities and differences among brain metastases from various origins. For example, our study revealed that patients with brain metastases from liver cancer, lung cancer, colorectal cancer, and gastric cancer face the highest mortality risks, consistent with earlier research findings (30–32). This indicates the need for increased vigilance when treating patients with brain metastases from these primary sites. The survival differences in brain metastasis patients based on primary tumour location may be due to genetic differences associated with different primary tumour sites and types (33). For example, third-generation tyrosine kinase inhibitors, such as Osimertinib, have shown exceptional efficacy in lung adenocarcinoma patients with brain metastases and alterations in the EGFR or ALK genes (34). Likewise, for patients with HER2-positive breast cancer who progress to CNS involvement after initial local treatment, various HER2-directed therapies have been shown to provide the possibility of long-lasting responses in some cases (35, 36). Additionally, randomized phase II data suggest that dual immune checkpoint inhibition using ipilimumab and nivolumab could be a promising treatment option for patients with brain metastases from melanoma (37).

Furthermore, among the different types of brain metastases, those originating from lung cancer are the most prevalent, a finding that aligns with results from other research studies (38). Unfortunately, the prognosis for brain metastases from lung cancer is also poor. This highlights the necessity for enhanced surveillance of the risk of brain metastases in lung cancer patients, particularly those aged 65 and above. Furthermore, our study revealed that the mortality risk for lung cancer patients with brain metastases increases significantly after the age of 65. A similar age-related risk pattern is observed in brain metastases from both breast cancer and lymphoma. Surgery is a critical treatment option for patients with brain metastases. The decision to proceed with surgery is typically based on the size of the metastases and several other comprehensive factors. While it is widely believed that neurosurgical resection offers an OS benefit, some studies suggest that surgical resection significantly enhances OS and functional status compared to whole-brain radiotherapy in treating brain metastases (39, 40). However, it is generally believed that neurosurgical resection provides an OS benefit (41, 42). Some studies indicate that surgical resection is associated with significantly improved OS and functional status compared to whole-brain radiotherapy for treating brain metastases (43). In the present study, a stratified analysis was conducted to assess the survival benefits of surgery for each type of brain metastasis, focusing on different primary sites. The study results showed that most brain metastases responded positively to surgical intervention. However, patients with brain metastases from liver cancer, bladder cancer, or esophageal cancer did not demonstrate a significant survival benefit following surgery. This indicates that brain metastases from different primary sites may necessitate the implementation of bespoke, personalized treatment plans.

To enhance the standardized management and prognostic analysis of patients with brain metastases, we have diverged from earlier studies that developed distinct risk-scoring models for metastases originating from various primary sites (44–47). In contrast, the primary site of the brain tumour was included as a covariate in the model. This approach addresses the need for different risk prediction models for brain metastases from various origins and avoids the bias associated with treating all brain metastases as a homogeneous category (48). In conclusion, although our study provides valuable epidemiological insights into prognostic factors influencing survival in brain metastases, several limitations—such as the retrospective design, data quality constraints, lack of detailed treatment information, and absence of molecular profiling—highlight the need for well-designed prospective studies. Incorporating molecular and genetic determinants into predictive models represents an essential next step to improve risk stratification, treatment personalization, and overall clinical outcomes for patients with brain metastases.

### Limitations of the study

This study has several limitations that should be acknowledged. First, its retrospective design based on data from the SEER database may introduce selection biases and preclude establishing definitive causal relationships. Second, the quality and completeness of data within the SEER database are inherently limited, with certain variables potentially incomplete or missing, thus potentially impacting the accuracy and generalizability of our findings. Additionally, detailed treatment information, including specifics regarding chemotherapy, radiotherapy, and immunotherapy, was not available, which could significantly influence patient survival outcomes. Furthermore, since the SEER database predominantly includes data from specific geographic regions, the generalizability of our results may be restricted by variations in race, socioeconomic status, and healthcare access. Moreover, the study did not incorporate potentially important prognostic factors such as genetic biomarkers, molecular characteristics, treatment responses, nor did it account for recurrence or progression of brain metastases, which might limit the precision of the predictive model. The primary focus on short-term survival analysis, with limited medium- and long-term follow-up data, further restricts the assessment of long-term survival outcomes and quality of life. Finally, the absence of detailed analysis regarding variations in surgical techniques, timing of surgical intervention, and institutional expertise may impact the robustness of our conclusions. Therefore, future research through large-scale prospective cohort studies is essential to validate and enhance the predictive accuracy of our findings.

## Conclusions

The findings of our study indicate that brain metastases originating from different primary sites exhibit distinct survival patterns and age-specific mortality risks. Several clinical factors, including sex, age, primary cancer site, income level, race, and therapeutic interventions (e.g., surgery, chemoradiotherapy), were identified as significant prognostic factors influencing patient survival.Furthermore, we developed a stable, accurate, validated risk prediction model for brain metastases. Although our risk prediction model demonstrated stable and validated predictive performance, it remains limited by reliance on retrospective SEER database data, lacking detailed molecular markers, genetic profiles, and comprehensive treatment information. Future research should incorporate prospective cohort designs, integrate genetic and molecular biomarkers, and apply advanced analytical approaches to enhance the clinical applicability and accuracy of predictive models for patients with brain metastases.

## Data Availability

The datasets presented in this study can be found in online repositories. The names of the repository/repositories and accession number(s) can be found in the article/supplementary material.
